# The Portrayal of Psychosis and Delusion in Film History (1895–1930)

**DOI:** 10.1192/j.eurpsy.2025.206

**Published:** 2025-08-26

**Authors:** D. Henkel

**Affiliations:** Institute for the History of Medicine and Medical Ethics, University of Cologne, Cologne, Germany

## Abstract

**Abstract:**

The turn of the century was an important era for the field of psychiatry. Influential physicians such as Sigmund Freud (1856-1939) or Eugen Bleuler (1857-1939) made headlines and new theories on the pathogenesis of psychological disorders emerged with the psychoanalytic approach – the whole field seemed in a state of transition. But did this modern image correspond to how two of psychiatry’s most famous conditions – psychoses and delusions – where framed in the mass media of film? Surprisingly, there are no systematic works on these question, neither by medical nor film historians. This lecture tries to close this gap and, for the first time in international research, provides a systematic overview of the representation of the theme in silent cinema. With the aim of sketching a representative image, 36 works portraying psychoses and / or delusions were identified and, among other things, historically classified, analyzed and evaluated for medical correctness. In summary, it is shown that the early film depicts the condition in a highly ambivalent way, from dangerous and untreatable Insanity to curable illness, thereby reflecting one of the most significant transitions in the history of the understanding delusional symptoms. The presented therapy methods – e. g. psychoanalysis, hypnotherapy, electrotherapy, water therapy and even music therapy –, thus rarely utilized, appear highly modern, while the depiction of the patients seems more influenced by the shadows of the past. Accompanied by scenes from representative works such as *The Other* (1913), *Wolf Blood* (1925), *Shadows* (1923) or the German classic *The Cabinet of Dr. Caligari* (1920), the presentation aims to illustrate all these characteristics of the early **‘**psychosis und delusion film’ and explores the important role of cinema as a source of medical history.

**Disclosure of Interest:**

None Declared

